# LPS exacerbates functional and inflammatory responses to ovalbumin and decreases sensitivity to inhaled fluticasone propionate in a guinea pig model of asthma

**DOI:** 10.1111/bph.13080

**Published:** 2015-03-24

**Authors:** A P P Lowe, R S Thomas, A T Nials, E J Kidd, K J Broadley, W R Ford

**Affiliations:** 1 Cardiff School of Pharmacy, Cardiff UniversityCardiff, UK; 2 Discovery Biology, Respiratory Centre of Excellence for Drug Discovery, GlaxoSmithKline Medicines Research CentreStevenage, UK

## Abstract

**Background and Purpose:**

Asthma exacerbations contribute to corticosteroid insensitivity. LPS is ubiquitous in the environment. It causes bronchoconstriction and airway inflammation and may therefore exacerbate allergen responses. This study examined whether LPS and ovalbumin co-administration could exacerbate the airway inflammatory and functional responses to ovalbumin in conscious guinea pigs and whether these exacerbated responses were insensitive to inhaled corticosteroid treatment with fluticasone propionate (FP).

**Experimental Approach:**

Guinea pigs were sensitized and challenged with ovalbumin and airway function recorded as specific airway conductance by whole body plethysmography. Airway inflammation was measured from lung histology and bronchoalveolar lavage. Airway hyper-reactivity (AHR) to inhaled histamine was examined 24 h after ovalbumin. LPS was inhaled alone or 24 or 48 h before ovalbumin and combined with ovalbumin. FP (0.05–1 mg·mL^−1^) or vehicle was nebulized for 15 min twice daily for 6 days before ovalbumin or LPS exposure.

**Key Results:**

Ovalbumin inhalation caused early (EAR) and late asthmatic response (LAR), airway hyper-reactivity to histamine and influx of inflammatory cells into the lungs. LPS 48 h before and co-administered with ovalbumin exacerbated the response with increased length of the EAR, prolonged response to histamine and elevated inflammatory cells. FP 0.5 and 1 mg·mL^−1^ reduced the LAR, AHR and cell influx with ovalbumin alone, but was ineffective when guinea pigs were exposed to LPS before and with ovalbumin.

**Conclusions and Implications:**

LPS exposure exacerbates airway inflammatory and functional responses to allergen inhalation and decreases corticosteroid sensitivity. Its widespread presence in the environment could contribute to asthma exacerbations and corticosteroid insensitivity in humans.

## Tables of Links

**Table:** 

TARGETS	
**GPCRs**^a^	**Enzymes**^d^
α1-adrenoceptor	Inducible (i)NOS
**Nuclear hormone receptors**^b^	
Glucocorticoid receptor	
**Catalytic receptors**^c^	
TLR4	

**Table:** 

LIGANDS	
Dexamethasone	IL-17
Fluticasone propionate (FP)	LPS
Histamine	LTC4
IFN-γ	Nitric oxide (NO)
IL-8 (CXCL8)	Noradrenaline
IL-13	TXA2

These Tables list key protein targets and ligands in this article which are hyperlinked to corresponding entries in http://www.guidetopharmacology.org, the common portal for data from the IUPHAR/BPS Guide to PHARMACOLOGY (Pawson *et al*., 2014) and are permanently archived in the Concise Guide to PHARMACOLOGY 2013/14 (*^a,b,c,d^*Alexander *et al*., 2013a,b,c,d).

## Introduction

Asthma exacerbations are worsening of respiratory symptoms characterized by increased airflow obstruction, mucus and oedema (Dougherty and Fahy, 2009). LPS from Gram-negative bacteria, also found ubiquitously in the environment in textiles, milk, tobacco smoke and particulate air pollution, is an important contributor to asthma exacerbations. LPS exposure evokes persistent bronchoconstriction (Michel *et al*., 1989) and can modify the early (EAR) and late asthmatic responses (LAR) of asthmatics to allergen challenge, with more neutrophils than seen with allergen alone (Hunt *et al*., 1994). A significant increase in eosinophil and neutrophil cell counts (Eldridge and Peden, 2000) and of airway hyper-responsiveness (AHR) in asthmatics (Boehlecke *et al*., 2003) was observed following combined dust mite and LPS challenges. Associated with neutrophilia are increases in cytokines such as IL-8 and IL-17 (Doe *et al*., 2010).

Paradoxically, LPS is also protective, with early life exposure decreasing allergy in later life (von Mutius, 2001). High doses (10–50 μg·mL^−1^) of LPS attenuate the development of allergic responses to ovalbumin (Ova) when administered during or close to allergen challenge (Tulić *et al*., 2000); high doses (1–20 μg·mL^−1^) of LPS have been demonstrated not only to suppress allergen-induced AHR and neutrophilic inflammation (Rodríguez *et al*., 2003; Delayre-Orthez *et al*., 2004; Murakami *et al*., 2007) but also to decrease AHR and inflammation (Tulic *et al*., 2002). In contrast, low doses (0.001–0.1 μg·mL^−1^) of LPS promote allergy when used as an adjunct to sensitization (Eisenbarth *et al*., 2002) or when administered during Ova challenge (Delayre-Orthez *et al*., 2004). However, higher doses of LPS have also been demonstrated to increase pulmonary inflammation (Komlósi *et al*., 2006) while attenuating functional responses to allergens (Vannier *et al*., 1991). LPS combined with Ova challenge can both increase and decrease mucus secretion depending on the LPS dose (Dong *et al*., 2009).

Inhaled corticosteroids are a frontline therapy in asthma treatment and are very effective at diminishing inflammatory and functional responses to allergen (Palmqvist *et al*., 2005). In a guinea pig model mimicking human asthma (Ricciardolo *et al*., 2008), the inhaled corticosteroid fluticasone propionate (FP) abolished the LAR, suppressed AHR and significantly reduced eosinophils (Evans *et al*., 2012). Subsets of asthmatic patients demonstrate decreased sensitivity to inhaled corticosteroids (Szefler *et al*., 2002). The origin of corticosteroid insensitivity is unknown, but mechanisms related to changes induced by LPS may be involved. Corticosteroid-insensitive asthmatics demonstrate inflammatory patterns characteristic of LPS activation, coinciding with high levels of LPS in lavage fluid (Goleva *et al*., 2008). Additionally, LPS alone induces neutrophilic inflammation that is unresponsive to inhaled corticosteroid treatment in healthy volunteers and asthmatics (Trapp *et al*., 1998; Michel *et al*., 2000). In animal models, the effect of combining allergen challenge with LPS on corticosteroid sensitivity has only been evaluated in one study using systemically administered dexamethasone (Komlósi *et al*., 2006). Moreover, LPS effects on the early and LARs have not been assessed in animal models. Thus, LPS appears to offer a means of altering the sensitivity of allergen-induced inflammation to corticosteroids and provide an animal model of steroid insensitivity. In the present study, we examine the hypothesis that LPS exposure modifies the functional and inflammatory responses of guinea pigs to allergen. Because the precise effects of LPS exposure on allergen responses appear to vary, we first examined the timing and number of LPS exposures upon the functional and inflammatory responses to Ova in sensitized guinea pigs. In view of the inconsistent literature data on whether high or low doses enhanced or suppressed the allergen responses, we chose a relatively high dose (30 μg·mL^−1^) for these studies.

## Methods

All chemicals were obtained from Sigma-Aldrich, Gillingham, Dorset, UK, or Fisher-Scientific, Loughborough, UK, unless stated otherwise.

### Animal husbandry

Male Dunkin-Hartley guinea pigs (total 134, 200–300 g; Charles River, Sulzfeld, Germany) were housed in pathogen-free conditions with 12 h light/dark cycles and allowed water and laboratory chow *ad libitum*. The total number of animals used was 134. They were provided with environmental enrichment with cardboard tubes and hay. All procedures were carried out in accordance with the Animals (Scientific Procedures) Act of 1986 and underwent ethical review by Cardiff University Biological Standards Committee. The ARRIVE guidelines on Animal Research: Reporting *In Vivo* Experiments have been adhered to in the design and execution of the study (Kilkenny *et al*., 2010; McGrath *et al*., 2010).

### Ovalbumin sensitization

Guinea pigs were sensitized by bilateral i.p. injection of Ova (VWR, Lutterworth, Leics, UK; technical grade 98.8% purity, 150 μg) and Al(OH)_3_ (100 mg) in 1 mL of normal saline on days 1, 4 and 7.

### Inhalation challenge with Ova and LPS

**Figure 1:**
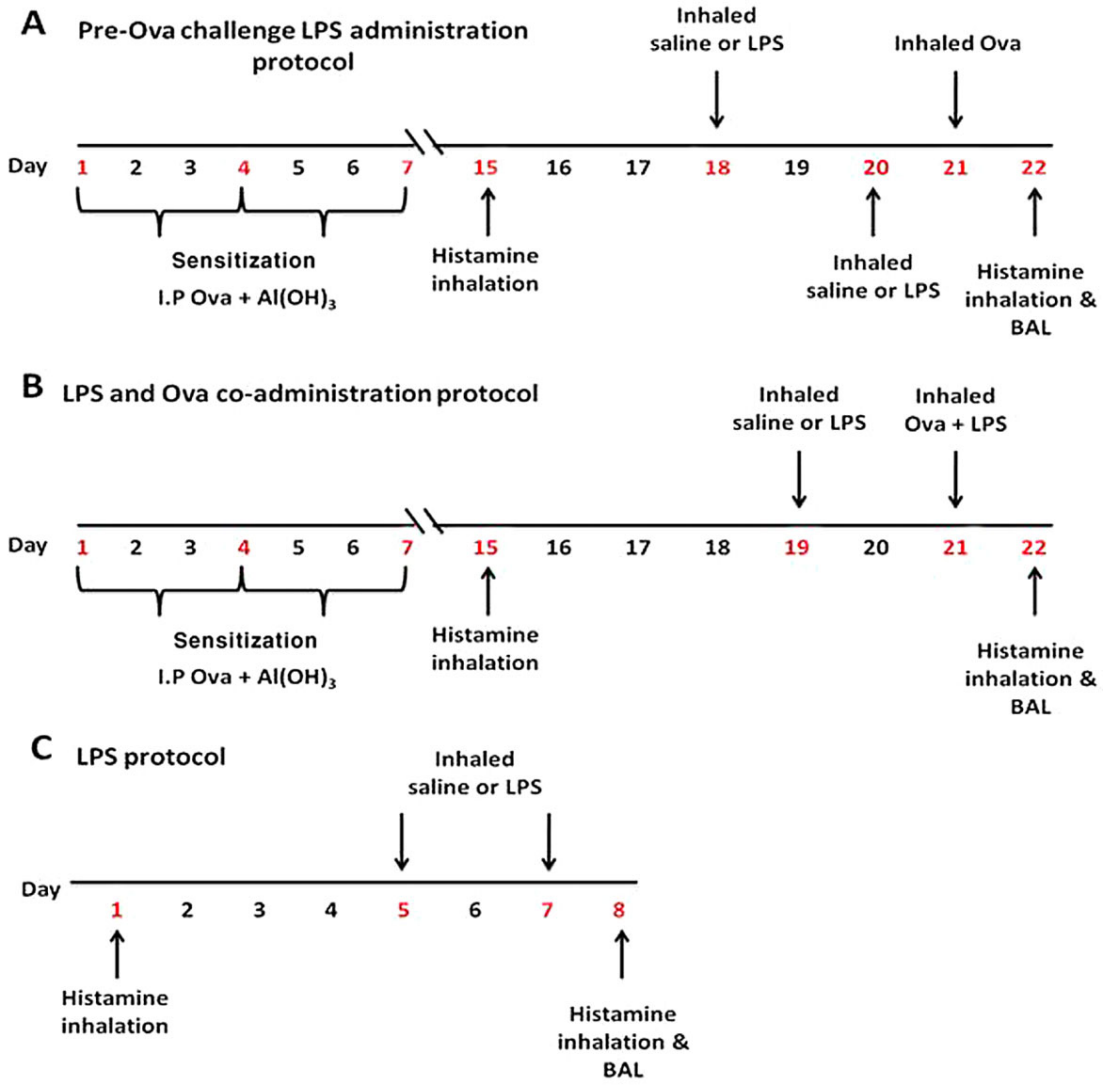
Ova and LPS protocols used in the present study. (A) Pre-Ova challenge LPS administration. (B) LPS and Ova co-administration. (C) LPS without Ova protocol.

Sensitized guinea pigs were exposed to inhaled Ova (300 μg·mL^−1^) on day 21. LPS (30 μg·mL^−1^) or normal saline exposure was by two protocols: 72 and 24 h pre-Ova exposure (Figure 1A), 48 h pre-Ova and co-administered with Ova (Figure 1B). Non-sensitized guinea pigs were exposed to LPS (30 μg·mL^−1^) or saline on days 5 and 7 (Figure 1C). Ova, LPS or saline was delivered into a Perspex exposure chamber (15 × 30 × 15 cm) for 1 h using a DeVilbiss (Somerset, Pennsylvania, PA, USA) nebulizer at 0.3 mL·min^−1^ and pressure of 20 lb p.s.i.

### Measurement of lung function

Airway function was measured as specific airway conductance (sGaw) in conscious, spontaneously breathing guinea pigs by non-invasive double-chamber plethysmography (Buxco Systems, Wilmington, NC, USA) (Now Data Sciences International, The Netherlands). Lung function was recorded following the final Ova, LPS or saline challenges at intervals for 12 and at 24 h.

Airway responses to aerosolized histamine were determined pre- and 24 h post-Ova or the final LPS/saline challenge using whole body plethysmography. Histamine (0.3 mM, 2 min) was delivered by the use of a Buxco nebulizer chamber at 0.5 L·min^−1^ and 10% duty (% every 6 s of nebulizing) per chamber. This nebulizer protocol evokes small non-significant bronchoconstriction in naïve guinea pigs. Lung function was measured before histamine inhalation and at 0, 5 and 10 min afterwards.

### Pulmonary inflammation and cytokines

Following the final histamine challenge, guinea pigs were administered an overdose of sodium pentobarbitone (Euthatal, 400 mg·kg^–1^ i.p.). Bronchoalveolar lavage was performed using normal saline (1 mL per 100 g) instilled for 3 min through a polypropylene cannula inserted into the trachea. This process was repeated, the samples pooled and the total number of cells·mL^–1^ counted using a Neubauer haemocytometer (Sigma-Aldrich). Differential cell counts (eosinophils, macrophages, lymphocytes and neutrophils) were performed after centrifuging 100 μL of lavage fluid onto glass microscope slides using a Shandon Cytospin (ThermoFisher Scientific, Hemel Hempstead, UK) at 110 x *g* for 7 min. Slides were subsequently stained with 1.5% Leishman's solution in 100% methanol for 6 min. A minimum of 200 cells were counted. Protein content in lavage fluid was determined as a measure of airway oedema by use of the BCA protein assay (Thermo Scientific Pierce Protein Biology, Cramlington, Northumberland, UK). One hundred milligrams of right middle lung lobe was homogenized in 1 mL lysis buffer (Thomas *et al*., 2006) using a Precellys® 24 tissue homogenizer (Stretton Scientific Ltd, Stretton, Derbyshire, UK) for 3 × 20 s cycles at 5500 r.p.m. Levels of IL-8, IL-17 and IL-13 were measured in diluted homogenized lung samples using human antibody elisa kits (R&D Systems, Abington, UK) once cross-reactivity was established. The limit of detection for IL-13 was 47 pg·mL^−1^, for IL-17 was 16 pg·mL^−1^ and for IL-8 31 pg·mL^−1^. Cytokine levels were adjusted for total lung protein and expressed as pg·mg^−1^ lung protein.

### Histology

Lung lobes were stored in formaldehyde and 1–2 mm bilateral sections were cut and dehydrated in increasing concentrations of alcohol and then chloroform. Tissue sections were set into paraffin wax blocks, 5 μm sections were cut using a microtome and fixed to poly-L-lysine-coated slides. Sections were deparaffinized with Histoclear® (Fisher Scientific) (3 × 5 min periods), rehydrated through graded ethanol (100% 2 × 3 min, 90% 3 min and 70% 3 min), washed under running tap water rinsed with distilled water and stained with Mayer's haematoxylin (2 min). Slides were then rinsed with water and stained with 1% eosin (90 s) before dehydration with graded ethanol and clearing with Histoclear (3 × 5 min periods) followed by air drying for 24 h and mounting with Histomount® (Life Technologies, Paisley, UK) and applying a coverslip. A semi-quantitative scoring method was applied to two airways per animal for assessment of general lung morphology by a blinded observer. 0 = normal lung; 1 = minimal peribronchiolar (PB) inflammation; 2 = slight inflammation in PB area; 3 = moderate PB inflammation; 4 = marked PB inflammation and cuffing (thickening), slight loss of lung structure; 5 = severe PB inflammation, cuffing and infiltration, loss of lung structure (Barends *et al*., 2004). Sections were also stained with Alcian blue/periodic acid Schiff (Ab/PAS) for assessment of goblet cell numbers in airway epithelium (Evans *et al*., 2012). After the sections were deparaffinized with Histoclear, rehydrated through graded alcohol and rinsed with water as described earlier, they were stained with 1% Alcian blue dissolved in 3% aqueous acetic acid (pH 2.5, 5 min) followed by washing under running tap water (5 min) and then with distilled water (5 min). Sections were then stained with periodic acid (0.5%, 5 min), rinsed with water and Schiff's reagent added (10 min) before a further rinse under running tap water and staining with Mayer's haematoxylin (20 s). Finally, they were rinsed with water (5 min), dehydrated through graded ethanol, cleared with Histoclear (3 × 5 min periods) followed by air drying before Histomount and then a coverslip were applied.

### Drug administration

FP (0.5 or 1 mg·mL^−1^) or vehicle [ethanol (30%), DMSO (30%) and saline (40%)] was nebulized for 15 min twice daily into an in-house Perspex exposure chamber using a DeVilbiss nebulizer from days 16 to 21 in Ova protocols and days 2 to 7 in LPS-only protocols. Two additional FP doses (0.05 and 0.1 mg·mL^−1^) were used in the Ova alone protocol.

### Data analysis

Lung function data were plotted as a percentage of baseline to take into account the individual differences in guinea pig baseline sG_aw_ values. To account for differences in the timing and duration of allergen responses, sG_aw_ was also expressed as the peak bronchoconstriction during the early (0–6 h) and late (6–12 h) phases and as AUC. The duration of the EAR was analysed as the time to return to 50% of peak EAR sGaw values. Results are plotted as the mean ± SEM. Student's *t*-tests were used to compare groups or data points. One-way anova followed by Dunnett's *post hoc* test were used when two or more groups were compared with a control group. *P*-values less than 0.05 were considered significant. A non-parametric Kruskal–Wallis anova test was used to analyse IL-8 levels, which were not detected in all samples.

## Results

### Effect of LPS on Ova-induced functional responses

**Figure 2:**
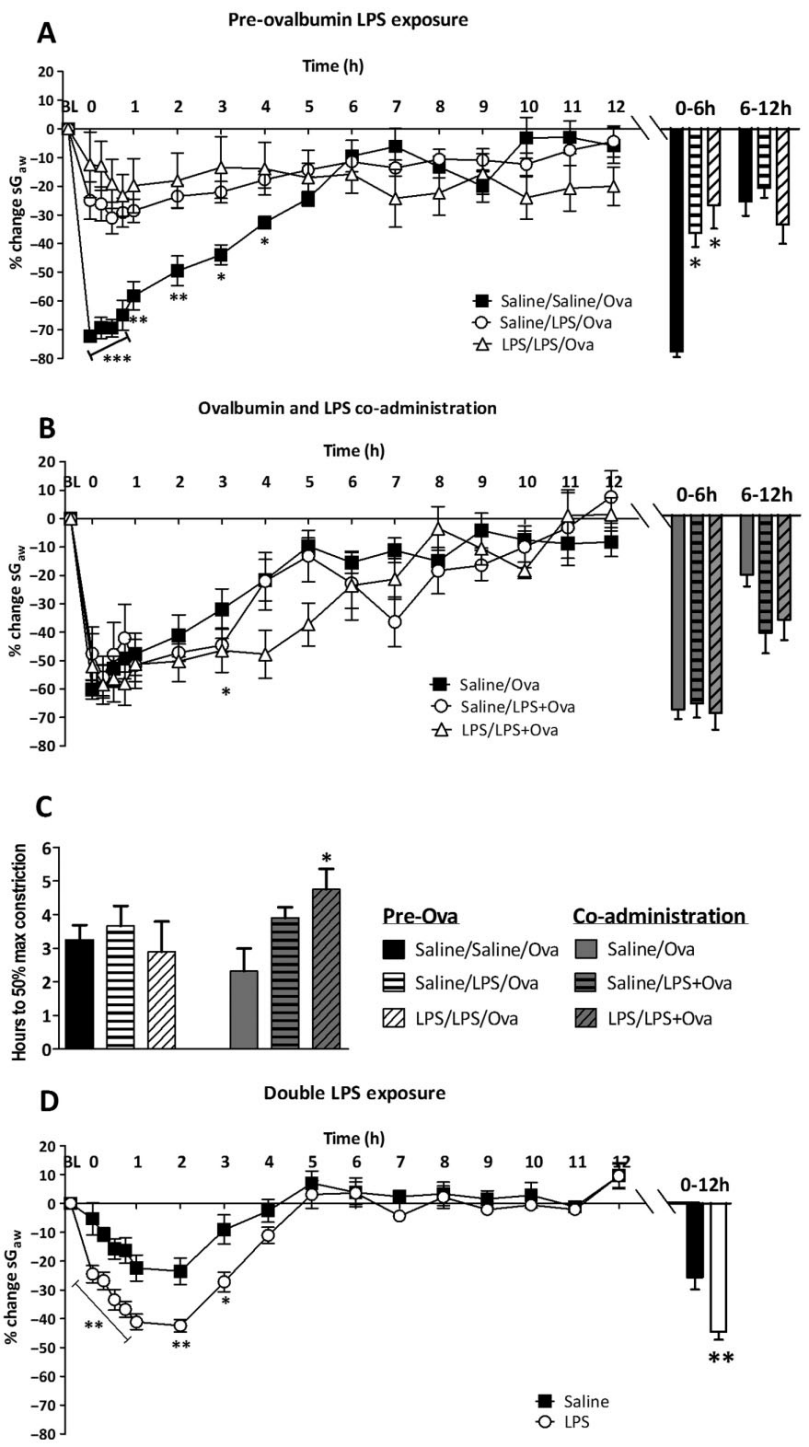
The mean time courses for changes in sGaw following Ova challenge of Ova-sensitized guinea pigs. Guinea pigs were treated with either saline or 30 μg·mL^−1^ LPS for 1 h, (A) 72 h and 24 h pre-Ova challenge, or (B) 48 h before Ova challenge and co-administered with Ova. Histograms represent the maximum bronchoconstriction values recorded during the EAR (0–6 h) and LAR (6–12 h). (C) Analysis of the time taken for EAR to recover to 50% of peak bronchoconstriction values. (D) Mean time courses for changes in sGaw following the second of two LPS or saline exposures in non-sensitized guinea pigs. Mean changes in sGaw are expressed as mean ± SEM percentage change from baseline prior to Ova challenge. *n* = 5–10. *Significantly different from respective naïve or Ova challenge and double-saline treatment group *P* < 0.05, **P < 0.01, ***P < 0.001; performed with one-way anova followed by Bonferroni post-test.

Ova challenge with saline pretreatment (saline/saline/Ova) induced distinct EAR (−73.5 ± 1.9%) and LAR (−24.0 ± 4.7%) (Figure 2A). A single LPS exposure, 24 h before Ova, and two LPS exposures, 72 and 24 h before Ova, attenuated the early phase, significantly decreasing the peak EAR to −34.4 ± 4.6 and −25.2 ± 27.7% respectively (Figure 2A). The LAR was unchanged by the two LPS pretreatments. In co-administration protocols, neither a single LPS exposure co-administered with Ova (saline/LPS + Ova) nor two LPS treatments, one administered 48 h before Ova challenge and one co-administered with Ova (LPS/LPS + Ova), altered either the peak EAR or LAR (Figure 2B). However, the latter protocol did increase the duration of the EAR (4.8 ± 0.6 h compared with saline 2.3 ± 0.7 h) (Figure 2C). This effect was not seen in pre-Ova LPS exposure protocols. At 24 h post-Ova sG_aw_ values had returned to baseline. LPS alone evoked a significant immediate bronchoconstriction peaking at 2 h (−41.3 ± 2.7% compared with saline −22.5 ± 4.5%) and returning to baseline after 5 h (Figure 2D).

### Effect of LPS on Ova-induced AHR

**Figure 3:**
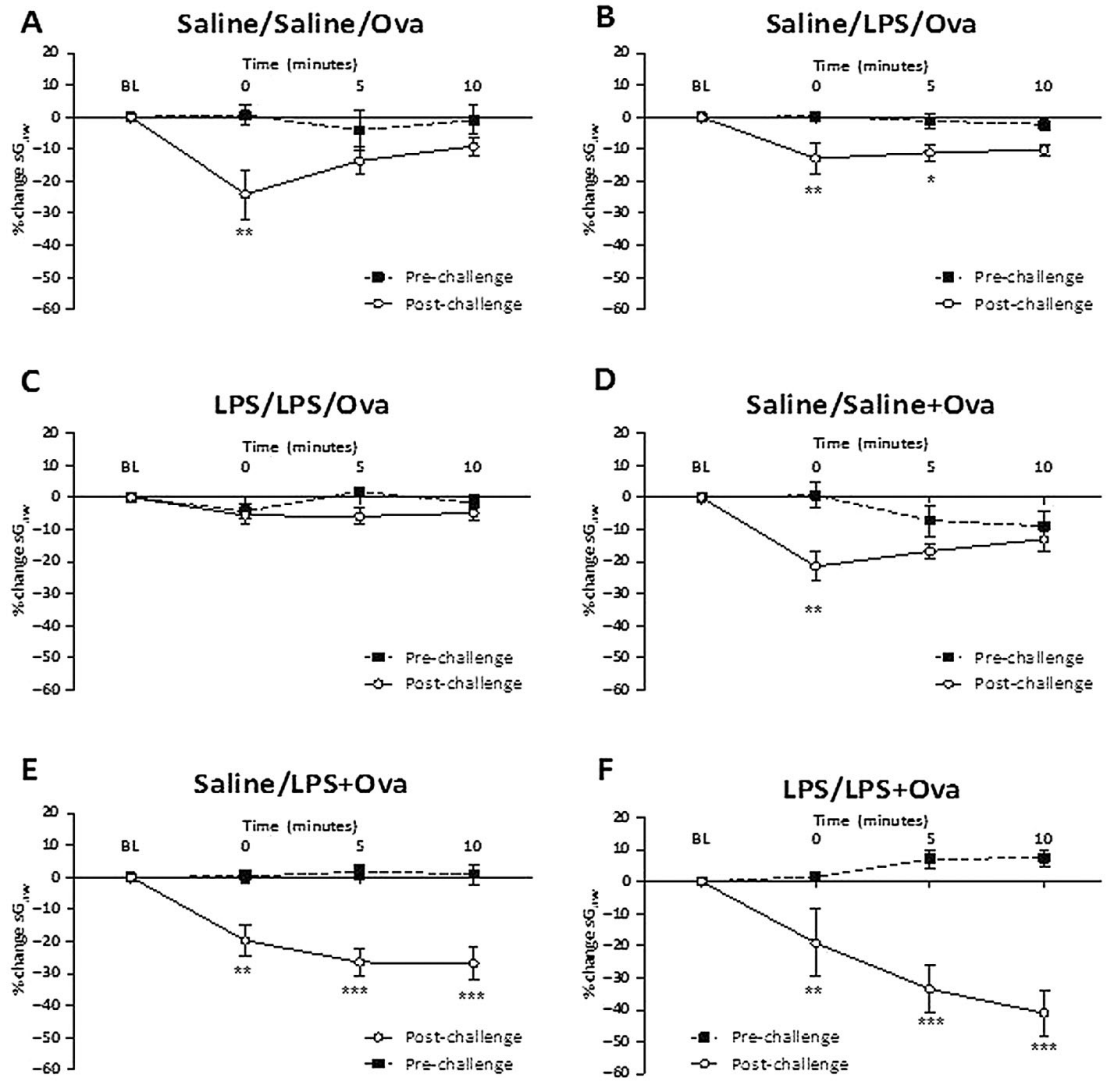
Response of the airways to nebulized histamine (0.3 mM, 2 min) before and 24 h after Ova challenge of Ova-sensitized guinea pigs. Guinea pigs were treated 72 and 24 h pre-Ova with: (A) saline twice, (B) saline and LPS (30 μg·mL^−1^), (C) LPS twice, or treated 48 h pre-Ova and with Ova challenge with (D) saline twice, (E) saline and LPS (F) LPS twice. Mean changes in sGaw are expressed as mean ± SEM percentage change from baseline. A negative value represents a bronchoconstriction. *n* = 5–10. *Significantly different from pre-Ova challenge values *P* < 0.05, **P < 0.01, ***P < 0.001; performed with a two-tailed *t*-test.

Ova challenge with no LPS treatment (saline/saline/Ova) significantly increased the bronchoconstriction to histamine compared with pre-challenge values, indicating the development of AHR (Figure 3A). With a single LPS exposure before Ova challenge (saline/LPS/Ova), there was still a significant increase in the histamine response (Figure 3B). However, two LPS exposures (LPS/LPS/Ova) abolished the development of AHR to histamine (Figure 3C). In the co-administration protocols (Figure 3E,F), Ova challenge with one LPS co-exposure (saline/LPS + Ova) or with two LPS exposures produced bronchoconstrictions to histamine. However, these bronchoconstrictions continued to increase over the next 10 min and were therefore more prolonged and significantly greater than in controls exposed to Ova and two saline challenges (Figure 3D). LPS alone increased the histamine bronchoconstriction at 0 min (−14.1 ± 3.8% compared with pre-LPS −0.9 ± 1.0%) and continued for 10 min (−16.1 ± 4.7% compared with pre-LPS −1.0 ± 2.5%) (not shown).

### Effect of LPS on Ova-induced pulmonary inflammation

**Figure 4:**
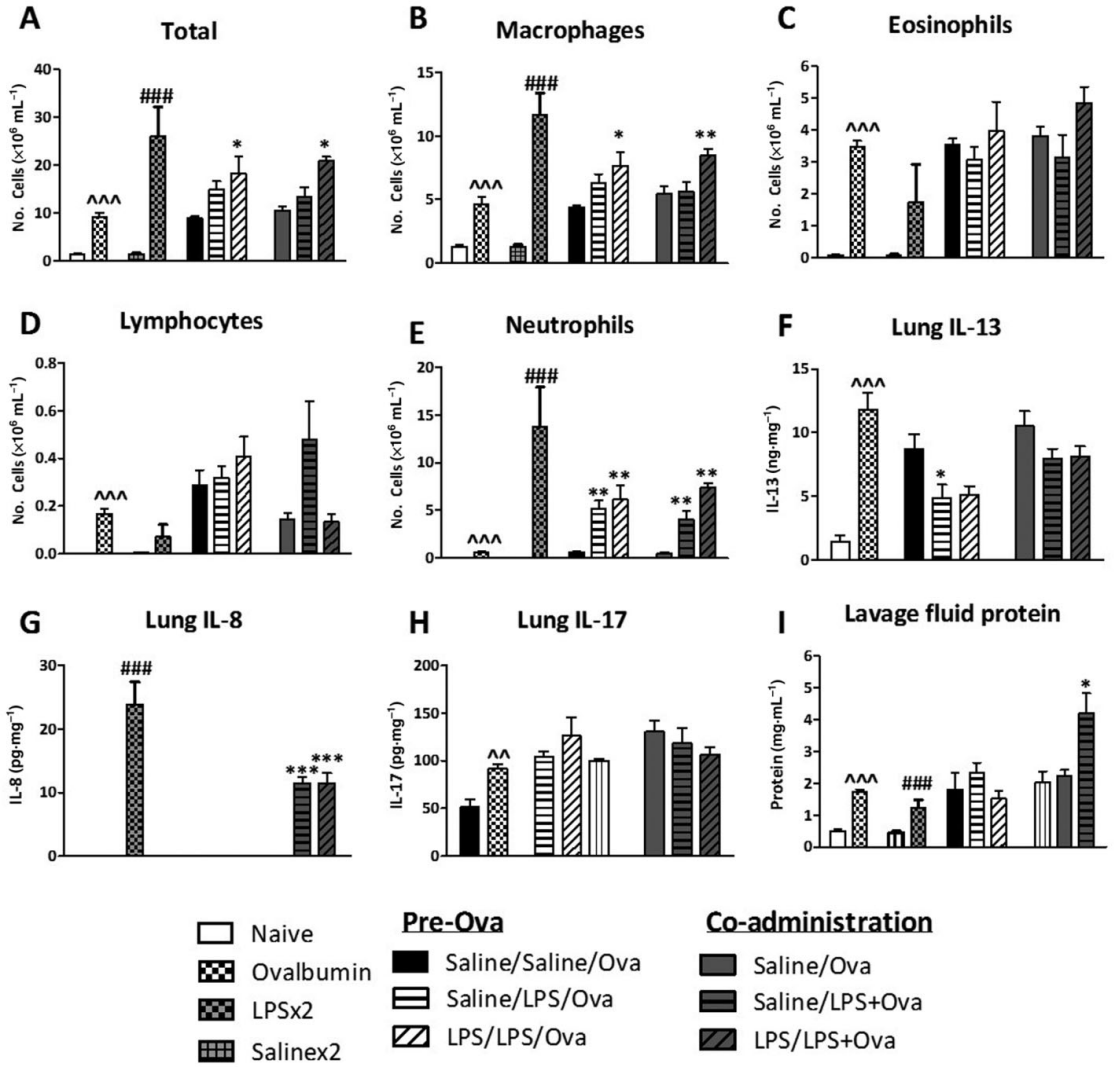
The total cell (A), macrophage (B), eosinophil (C), lymphocyte (D) and neutrophil (E) counts in bronchoalveolar fluid and IL-13 (F), IL-8 (G) IL-17 (H) in homogenized lung and lavage fluid protein levels (I) of Ova-sensitized guinea pigs determined 24 h after Ova challenge. Guinea pigs were treated with either saline or 30 μg·mL^−1^ LPS for 1 h either 72 and 24 h pre-Ova or 48 h before Ova challenge and co-administered with Ova. Naïve (unsensitized and challenged), Ova sensitized and challenged double-saline and double-LPS challenged guinea pigs are also shown. IL-8 was undetectable in Ova-sensitized and -challenged guinea pigs and was therefore analysed by Kruskal–Wallis non-parametric anova. Results are expressed as mean ± SEM; *n* = 5–10. *Significantly different from respective Ova challenge and double-saline treatment group (saline/saline/Ova) *P* < 0.05, **P < 0.01, ***P < 0.001; performed with one-way anova followed by Bonferroni post-test. ^^Significantly different from naive animals *P* < 0.01, ^^^*P* < 0.001. ^###^Significantly different from saline ×2 *P* < 0.001.

Ova challenge with no LPS exposure significantly increased total cells and all cell types in lavage fluid compared with naïve animals (Figure 4). Total cell numbers (Figure 4A) further increased with additional LPS exposure, reaching significance after two doses of LPS in both pre-Ova and co-administration protocols. This change mainly reflected increases in neutrophils (Figure 4E) occurring with two doses of LPS in both pre-Ova and co-administration protocols. Macrophages (Figure 4B) were also significantly increased with two doses of LPS in both the pre-Ova (LPS/LPS/Ova) and co-administration (LPS/LPS + Ova) protocols (Figure 4B). Eosinophils (Figure 4C) and lymphocytes (Figure 4D) were unchanged by LPS treatment compared with the effect of Ova alone. IL-13 levels (Figure 4F) increased significantly following Ova exposure compared with naïve animals. LPS exposure did not significantly increase IL-13 levels further in any protocol, although there was a significant reduction with a single pre-Ova exposure (saline/LPS/Ova). IL-8 (Figure 4G) was significantly increased in LPS and Ova co-administration groups exposed to one and two LPS exposures compared with Ova and saline. IL-17 levels (Figure 4H) significantly increased following Ova exposure compared with naïve animals. LPS treatment did not change IL-17 levels compared with Ova + saline in any protocol. Total lavage protein (Figure 4I) significantly increased after Ova challenge alone compared with naïve animals. Only with two LPS exposures in the co-administration protocol was there a further significant increase.

LPS alone (two exposures 48 h apart) in non-sensitized guinea pigs significantly increased total cells compared with two exposures with saline (Figure 4A) as well as macrophages (Figure 4B) and neutrophils (Figure 4E). LPS also significantly increased IL-8 (Figure 4G) compared with saline treatment where it was undetectable. These levels of macrophages, neutrophils and IL-8 appeared to be higher than with the LPS/LPS + Ova combinations, although not significantly.

### Effect of Ova and LPS on airway histology and goblet cell meta/hyperplasia

**Figure 5:**
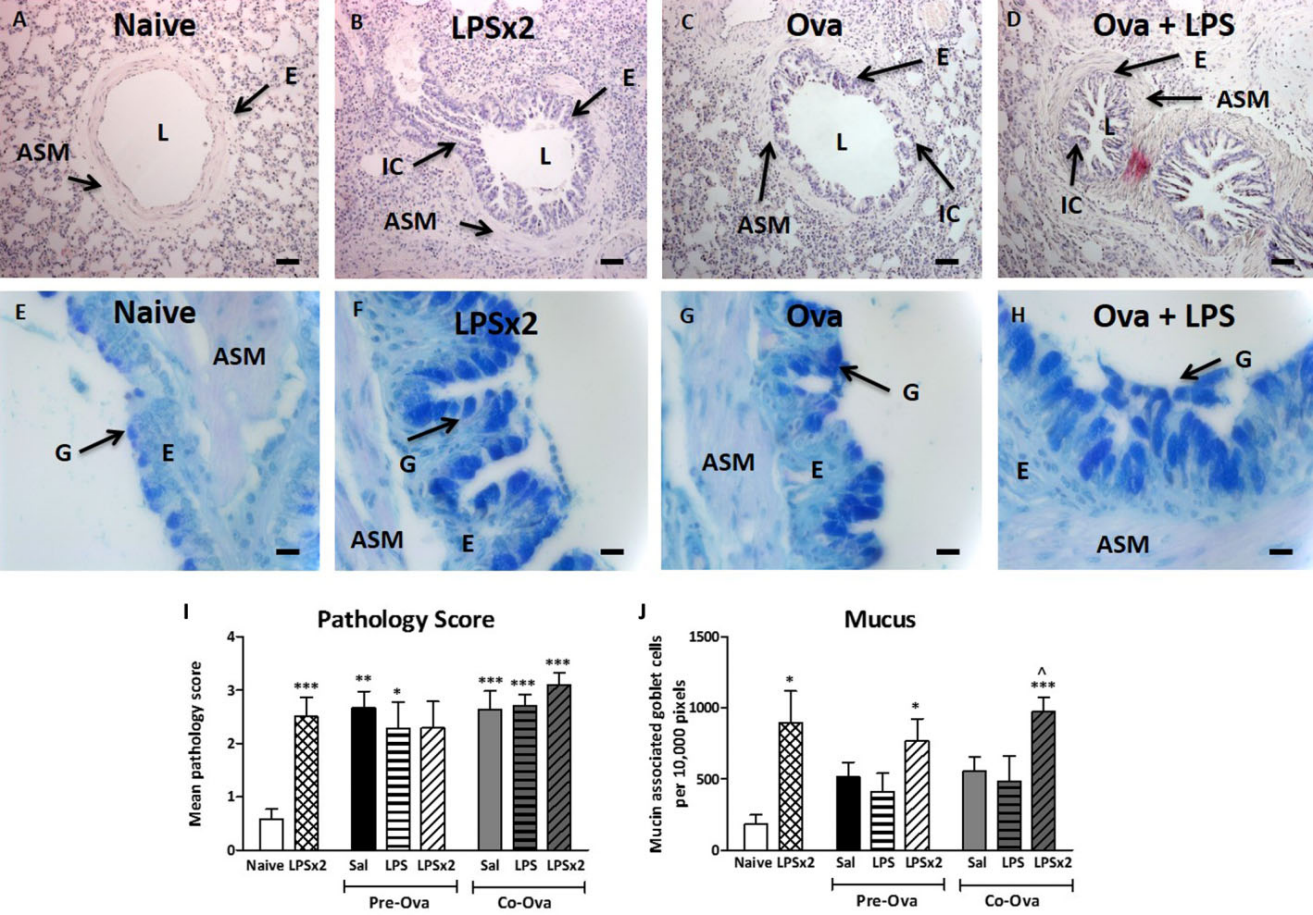
Bronchiolar changes and goblet cell staining in guinea pigs: naïve (A and E), double-LPS challenged (B and F), Ova sensitized and challenged treated with either saline (C and G) or LPS 48 h pre-Ova and co-administered with Ova (D and H). ASM, airway smooth muscle; E, epithelium; IC, inflammatory cell; G, goblet cell; L, lumen. A–D were stained with haematoxylin and eosin, original magnification 100× (bar = 25 μm). E–H with alcian blue/periodic acid Schiff, original magnification 400× (bar = 100 μm). (I) The mean pathology score of the PB area. (J) The number of mucin-associated goblet cells per 10 000 epithelial pixels. Results are expressed as mean ± SEM; *n* = 5–10. *Significantly different from respective naïve *P* < 0.05, **P < 0.01, ***P < 0.001 performed with anova followed by Tukey's post-test. ^Significantly different from saline control (saline/saline+Ova) *P* < 0.01.

Figure 5A–C shows lung sections stained with haematoxylin and eosin for assessment of general morphology. Ova challenge with saline treatment increased the staining for inflammatory cells in the peribronchiolar (PB) area (Figure 5C). LPS did not further change the inflammatory cell presence in the bronchiole in any protocol (LPS/LPS + Ova; Figure 5D). LPS alone significantly increased the mean pathology score compared with saline exposure. Figure 5E–H shows the lung sections stained with Alcian blue/periodic acid to reveal mucus-containing goblet cells. Guinea pigs treated with saline before Ova challenge (saline/saline/Ova) showed increased numbers of goblet cells (Figure 5G). Treatment with LPS and Ova further increased the number of goblet cells. Figure 5J represents the number of mucin-associated goblet cells per 10 000 pixels of epithelium. In the pre-Ova administration protocol, two exposures to LPS significantly increased goblet cells compared with naïve. In the co-administration protocol, LPS exposure before and co-administered with Ova (LPS/LPS + Ova) significantly increased goblet cells compared with naïve and to Sal/LPS + Ova. LPS alone significantly increased goblet cell numbers compared with saline.

### Sensitivity of functional responses to inhaled FP

**Figure 6:**
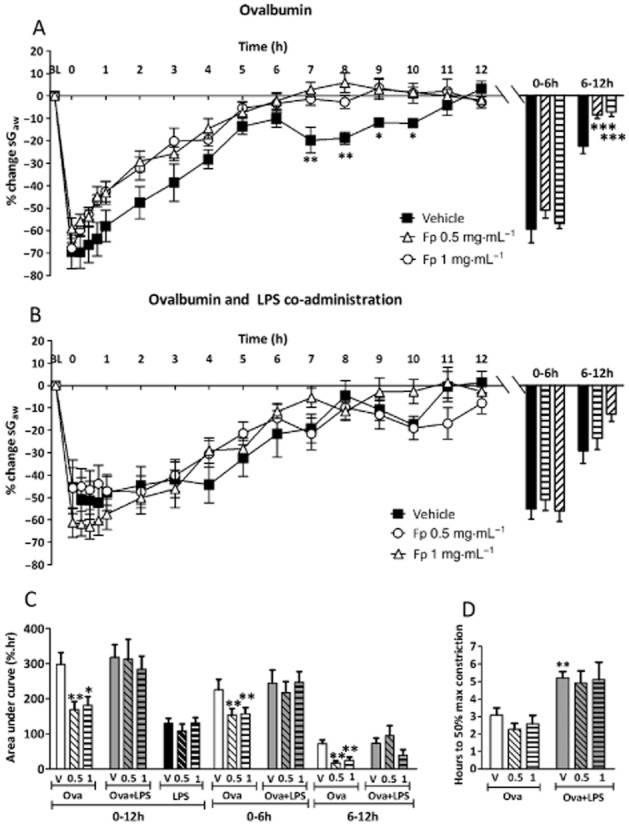
The mean time courses for changes in sGaw following (A) Ova challenge in sensitized guinea pig (B) treatment with LPS (30 μg·mL^−1^) 48 h pre-Ova and co-administered with Ova challenge in sensitized guinea pigs treated with either inhaled vehicle or FP (0.5 or 1 mg·mL^−1^), split into twice daily doses. The histogram represents the maximum bronchoconstriction values recorded during the EAR (0–6 h) and LAR (6–12 h). (C) AUC analysis of sGaw values for 0–12, 0–6 and 6–12 h post-Ova challenge. (D) Analysis of the time taken for EARs to recover to 50% of peak bronchoconstriction values after Ova and LPS co-administration. Mean changes in sGaw are expressed as mean ± SEM percentage change from baseline prior to Ova challenge. *n* = 5–10. *Significantly different from vehicle treatment *P* < 0.05, performed with anova followed by Dunnett's post-test.

The effects of FP were only examined in the LPS pre- and co-administration model. Ova challenge of vehicle-treated guinea pigs caused an EAR and LAR (Figure 6A). No decrease in the LAR was observed with 0.05 or 0.1 mg·mL^−1^ FP (data not shown). Treatment with 0.5 and 1 mg·mL^−1^ FP significantly attenuated the LAR. This was reflected in a decrease both in the 6−12 h peak response and in the AUC between 6 and 12 h for both 0.5 and 1 mg·mL^−1^ FP compared with vehicle (Figure 6C). The peak EAR (0−6 h) after Ova challenge was not inhibited by 0.5 and 1 mg·mL^−1^ FP, but the 0−6 h AUC was inhibited due to smaller bronchoconstrictions between 1 and 6 h. In the combined Ova and LPS challenge protocols (LPS/LPS + Ova; Figure 6B), a LAR and prolonged EAR were observed. The longer EAR was illustrated by the time to recover to 50% being significantly extended (Figure 6D). FP 0.5 mg·mL^−1^ treatment did not significantly alter the time course or attenuate the peak of the LAR. FP 1 mg·mL^−1^ appeared to reduce the LAR, but this was not significant when assessed from the 6−12 h AUC. Neither dose of FP significantly reduced the prolonged EAR response measured as the time to recover to 50% (Figure 6D). After LPS alone, vehicle-treated groups demonstrated a peak bronchoconstriction between 0 and 12 h, which was not significantly attenuated by FP treatment (Figure 6C).

### Sensitivity of AHR to inhaled FP

**Figure 7:**
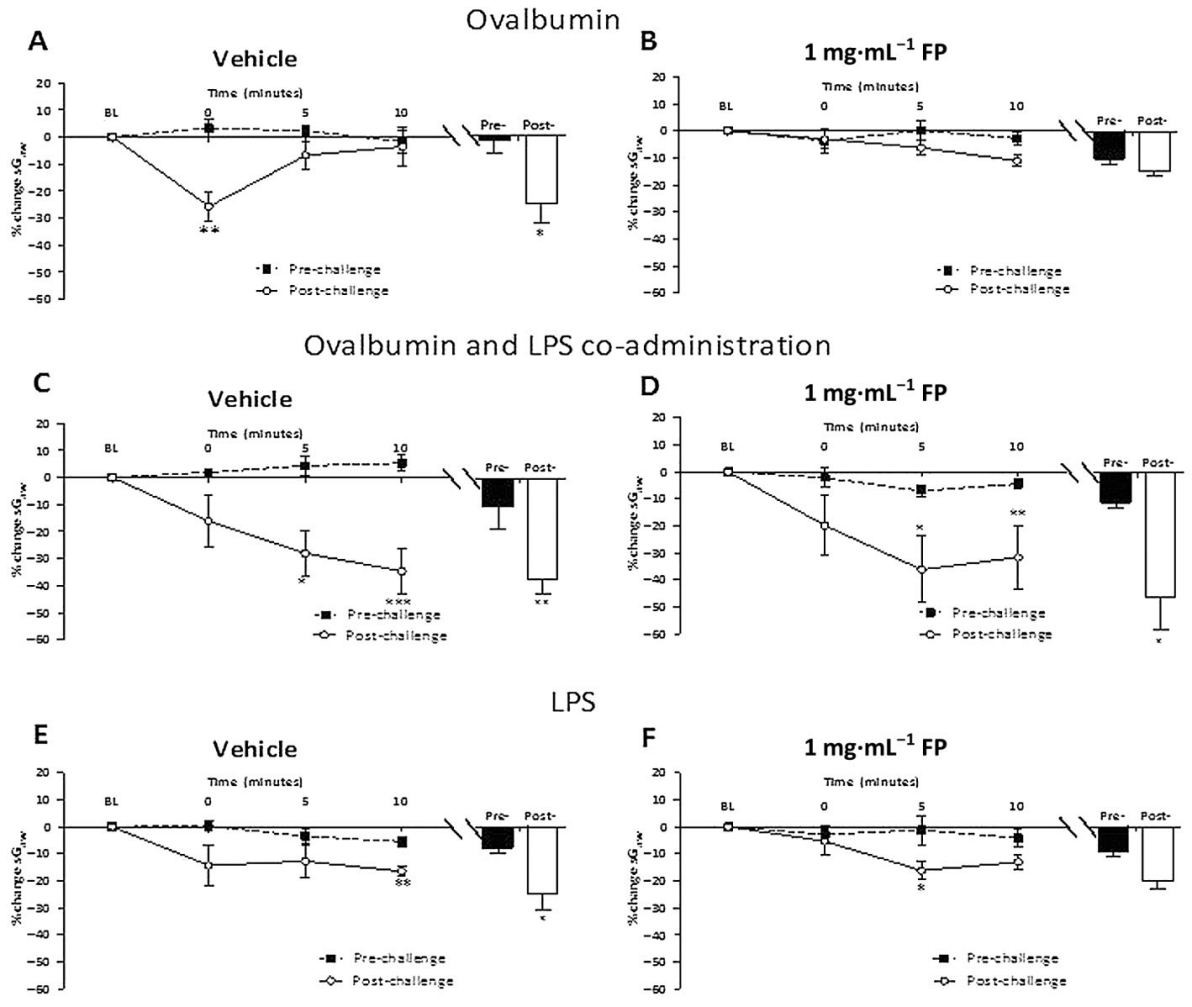
Response of the airways to nebulized histamine delivered in a plethysmograph (0.3 mM, 10% duty cycles and 0.5 L·s^–1^/chamber over 2 min) in guinea pigs (A) sensitized and challenged with Ova and treated with inhaled vehicle or (B) FP (1 mg·mL^−1^) split into twice daily doses or (C) sensitized and challenged with Ova treated with LPS (30 μg·mL^−1^) 48 h pre-Ova and co-administered with Ova and treated with inhaled vehicle or (D) FP (1 mg·mL^−1^) split into twice daily doses or (E) treated with LPS (30 μg·mL^−1^) twice and treated with inhaled vehicle or (F) FP (1 mg·mL^−1^), split into twice daily doses. The histogram represents the maximum bronchoconstriction values recorded during pre- and post-challenge histamine challenge. Values were recorded pre- and 24 h post-Ova challenge. Mean changes in sGaw are expressed as mean ± SEM percentage change from baseline. A negative value represents a bronchoconstriction. *n* = 5–10. *Significantly different from pre-Ova challenge values *P* < 0.05, **P < 0.01, ***P < 0.001; performed with a two-tailed *t*-test.

Ova challenge of vehicle-treated guinea pigs induced AHR, as a significant increase in the bronchoconstrictor response to histamine post-Ova compared with pre-Ova challenge (Figure 7A). Treatment with 0.05 and 0.1 mg·mL^−1^ FP did not attenuate this increased response. Treatment with 0.5 (data not shown) or 1 mg·mL^−1^ FP (Figure 7B) abolished the development of AHR. In Ova and LPS challenge protocols, vehicle-treated groups displayed a bronchoconstriction to histamine post-Ova challenge that increased across the 10 min of measurement (Figure 7C). FP 0.5 (not shown) and 1 mg·mL^−1^ treatment did not significantly alter this response with significant bronchoconstrictions still present at 10 min (Figure 7D). When challenged with LPS alone, vehicle-treated groups displayed a small increase in bronchoconstrictor response to histamine significant at 10 min compared with pre-challenge (Figure 7E). The peak response to histamine for the entire 10 min was significantly increased (−22.0 ± 5.2%) compared with pre-LPS responses (−7.0 ± 1.8%). Treatment with 0.5 (data not shown) and 1 mg·mL^−1^ FP did not abolish the development of AHR, with a significant increase in sG_aw_ at 5 min post-histamine compared with pre-LPS. However, the peak response to histamine was no longer significantly greater than the pre-challenge peak (Figure 7F).

### Sensitivity of inflammatory responses to inhaled FP

**Figure 8:**
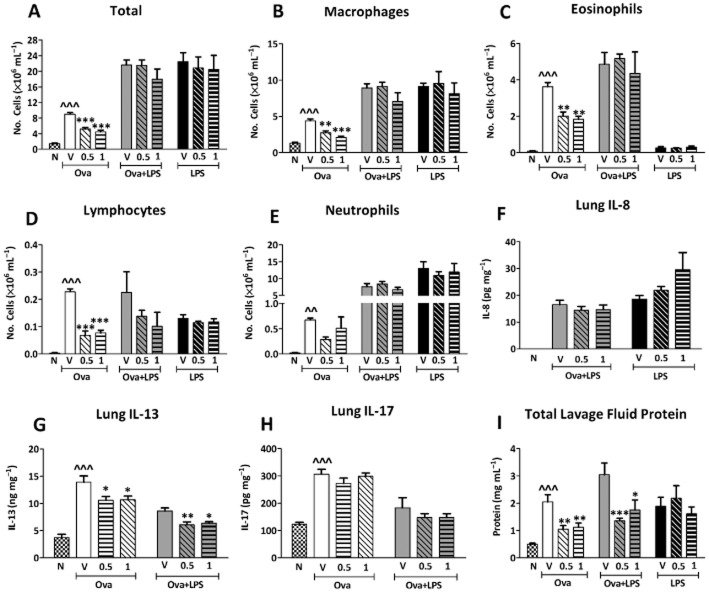
The total cell (A), macrophage (B), eosinophil (C), lymphocyte (D) and neutrophil (E) counts in the bronchoalveolar fluid and homogenized lung (F) IL-13, (G) IL-8 (H) IL-17 and lavage fluid protein levels (I) in guinea pigs sensitized and challenged with Ova, sensitized and challenged with Ova and treated with LPS (30 μg·mL^−1^) 48 h pre-Ova and co-administered with Ova or treated with LPS (30 μg·mL^−1^) twice. Guinea pigs were treated with either inhaled vehicle or FP (0.5 or 1 mg·mL^−1^) split into twice daily doses. Naïve (unsensitized and challenged) guinea pigs are also shown for comparative purposes only. Results are expressed as mean ± SEM; *n* = 5–10. *Significantly different from respective vehicle *P* < 0.05, **P < 0.01, ***P < 0.001; ^∧∧∧^Significantly different from naïve; performed with anova followed by Dunnett's post-test or by Kruskal–Wallis non-parametric test in the case of IL-8.

After Ova challenge, vehicle-treated groups displayed an increase in total inflammatory cells in lavage fluid (Figure 8A) that was significantly attenuated by treatment with 0.5 and 1 mg·mL^−1^ FP, but not by 0.05 and 0.1 mg·mL^−1^ FP (data not shown). Macrophages (Figure 8B), eosinophils (Figure 8C), lymphocytes (Figure 8D) and neutrophils (Figure 8E) were also significantly increased by Ova challenge in vehicle-treated groups. FP 0.5 and 1 mg·mL^−1^ FP significantly attenuated eosinophils, macrophages and lymphocytes. After Ova and LPS pre- and co-administrations (LPS/LPS + Ova), total and differential cell counts were not significantly reduced by 0.5 or 1 mg·mL^−1^ FP.

LPS alone also increased total cells, macrophages, lymphocytes and neutrophils but not eosinophils. These changes were not inhibited by 0.5 or 1 mg·mL^−1^ FP (Figure 8). IL-8 (Figure 8F) was not detectible in Ova alone groups (data not shown). In Ova and LPS (LPS/LPS + Ova) and LPS alone groups, 0.5 and 1 mg·mL^−1^ FP did not significantly attenuate IL-8. In Ova alone groups, IL-13 levels (Figure 8G) were significantly reduced by 0.5 and 1 mg·mL^−1^ FP compared with vehicle. In Ova and LPS co-administration groups, 0.5 and 1 mg·mL^−1^ FP also decreased IL-13 levels compared with vehicle. IL-17 (Figure 8H) levels were unchanged by 0.5 and 1 mg·mL^−1^ FP in Ova alone and Ova and LPS (LPS/LPS + Ova) protocols. Lavage fluid protein levels (Figure 8I) were significantly decreased by 0.5 and 1 mg·mL^−1^ FP after Ova challenge compared with vehicle in the LPS/Ova co-administration group (LPS/LPS + Ova), but not after LPS alone.

## Discussion

This study shows that LPS has a complex interaction with allergen-induced responses, attenuating and exacerbating them depending on the timing and number of LPS exposures. This is the first study to examine the effects of LPS on both inflammatory and functional responses to allergen challenge. It also demonstrates that LPS decreases the sensitivity of allergen-induced functional and inflammatory responses to inhaled FP, a mainstay of asthma management. As LPS is ubiquitous throughout the environment, this study highlights how LPS may be an important modifier of asthma severity and its response to inhaled corticosteroids.

### The effect of LPS on allergen-induced functional responses

Variable effects of LPS on allergen-induced functional responses have been reported previously. LPS can both decrease EARs (Vannier *et al*., 1991) and increase the time taken to reach peak early phase bronchoconstriction (Tulic *et al*., 2002). Similarly, LPS can diminish (Komlósi *et al*., 2006) or exacerbate (Rodríguez *et al*., 2003; Delayre-Orthez *et al*., 2004) allergen-induced AHR. This is the first reported study to directly compare the effects of the number and timing of LPS exposures around the time of allergen challenge on functional responses. We have demonstrated that LPS exposure 24 h before allergen challenge attenuates the EAR, whereas LPS co-administered with allergen leaves the early phase response unchanged. The addition of a second LPS exposure co-administered with Ova prolonged the EAR, an effect previously unreported. Concordantly, LPS exposure 24 h before allergen challenge diminished AHR to histamine, whereas LPS co-administered with allergen prolonged the bronchoconstrictor response to histamine.

The EAR is primarily mediated by allergen-induced release of mast cell-derived bronchoconstrictor mediators including histamine, LTC_4_ and TXA_2_ (Vannier *et al*., 1991). Mast cells can also degranulate in response to other stimuli such as LPS via toll-like receptor (TLR4) activation (Masuda *et al*., 2002; Murakami *et al*., 2007), which can inhibit the EAR following Ova challenge (Vannier *et al*., 1991). As the process of regenerating preformed mediators following degranulation can take up to 24 h (Dvorak, 2005), the timing of the LPS exposure may determine the amount of mediators mast cells have to release in response to allergen challenge. At 24 but not 48 h after LPS exposure mast cells may be largely still depleted, resulting in limited bronchoconstriction in response to allergen challenge. Attenuation of the EAR may also alter later responses to allergen such as the LAR and AHR that are initiated during the EAR (Smith and Broadley, 2007). This was observed in the present study where attenuation of the EAR was accompanied by diminished allergen-induced AHR. The LAR has been reported to be decreased in rats following allergen and LPS exposure (Tulic *et al*., 2002), although this decrease was only inferred from a decreased AHR. In humans, exposure to LPS 24 h before allergen challenge did not alter inflammatory or functional AHR responses to methacholine (Sohy *et al*., 2006). It is interesting that these authors used a concentration of LPS (50 μg·mL^−1^) similar to that used by ourselves (30 μg·mL^−1^) and considered that it was equivalent to the levels found in the environment.

LPS has the potential to potentiate the EAR by increased mast cell degranulation through enhanced IgE-induced cross-linking (Masuda *et al*., 2002). This may underlie the increased EAR duration in the allergen and LPS co-administration protocol. However, elongation of the EAR was only observed after the addition of a second dose of LPS, suggesting that priming is first required. Indeed, LPS-induced bronchoconstrictions increase with multiple exposures (Toward and Broadley, 2002), suggesting a priming action. LPS priming is mediated by up-regulation of TLR4 and downstream messengers in cells including neutrophils and macrophages (Lin *et al*., 2006; Hoogerwerf *et al*., 2010; Reino *et al*., 2012). Subsequent doses of LPS then evoke an increased release of bronchoconstrictive substances (Hoogerwerf *et al*., 2010).

Neutrophil- and macrophage-derived free radicals cause epithelial damage and reduce the activity of enzymes such as histamine *N*-methyltransferase important in the metabolism of histamine (Hoshi *et al*., 1996; Haddad, 2004). This could extend the biological *t*_1/2_ of histamine, prolonging histamine-induced bronchoconstriction, as observed with LPS and allergen co-administration in the present study. LPS also increases the production of NO, which can induce AHR (Strohmeier *et al*., 2001). However, the lack of activity of iNOS inhibitors at reducing AHR in other Ova and LPS combination models would suggest NO is not a critical mediator (Komlósi *et al*., 2006).

### The effect of LPS on allergen-induced inflammatory responses and histology

Allergen-induced inflammation is initiated during the EAR and increases significantly during the LAR, with a predominance of eosinophils and CD4+ lymphocytes observed (Nabe *et al*., 2005). Previous studies have reported that the addition of LPS exposure local to the respiratory tract at the same time or after Ova increases total inflammation and the predominance of neutrophils in both mice (Murakami *et al*., 2007) and rats (Tulić *et al*., 2000). Unlike functional responses to allergen, this effect was not related to the timing of LPS exposure in the present study, with no difference in the inflammation found between the two LPS protocols. However, the total number of cells and macrophages did increase with an increased number of LPS exposures. This agrees with a study by Jiang *et al*. (2006) who showed a transient increase in neutrophils following i.p. administration of LPS. But this contrasts with Rodríguez *et al*. (2003) who showed that LPS given simultaneously with allergen can also decrease total cell numbers and not increase neutrophilia when given systemically by i.v. injection. It was interesting to note that two exposures of LPS alone produced significant increases in cells particularly neutrophils and that these levels were greater than with the LPS/Ova combinations. It might be argued therefore that there was an inhibition between LPS and Ova. However, this can be discounted when comparisons are made between Ova alone and Ova + LPS where the latter cell levels are clearly significantly greater than with Ova alone.

Neutrophilia is facilitated by IL-8 and the IL-17 family of cytokines (Fogli *et al*., 2013). In particular, IL-17A and KC, the murine functional homologue for IL-8, have been demonstrated to be important drivers of increased neutrophilia in a house dust mite model (de Boer *et al*., 2012). However, their importance in Ova and LPS combination responses is relatively unknown. In the present study, increased neutrophilia in Ova and LPS co-administration protocols coincided with increased IL-8 but not IL-17A above that observed with Ova alone, suggesting the former is more important in this model. However, it does not discount other members of the IL-17 cytokine family being important. IL-8 is a transient cytokine with levels that fluctuate (Angrisano *et al*., 2010). This may be why IL-8 levels were undetectable in naïve animals despite increased neutrophils after the second LPS exposure.

Allergen challenge promotes a Th2 pattern of inflammation characterized by eosinophils and cytokines such as IL-13 (Komlósi *et al*., 2006). In asthma, an altered innate immunity response to infection and pollutants including LPS appears to have a role in amplifying the Th2-driven inflammatory cascade (Holgate, 2012). The effects of LPS on Th2 responses in animal models of allergy remain uncertain as LPS has been demonstrated to increase (Delayre-Orthez *et al*., 2004), decrease (Gerhold *et al*., 2002; Rodríguez *et al*., 2003; Komlósi *et al*., 2006) and not alter (Murakami *et al*., 2007) them. The timing of LPS exposure or the number of exposures do not appear to be important in determining the Th2 response as eosinophil and IL-13 levels were generally unchanged between protocols. A more crucial factor in the effect of LPS on Th2 responses is the dose of LPS. Studies reporting increased Th2 responses often use lower doses of LPS, whereas decreases are associated with high LPS doses, as used in the present study, which promote Th1 pro-inflammatory responses with increased IFN-γ (Gerhold *et al*., 2002; Delayre-Orthez *et al*., 2004).

Airway oedema is a feature of allergic airway inflammation and worsens during asthma exacerbations (Dougherty and Fahy, 2009). Airway oedema increased following allergen challenge as indicated in the present study by the increased lavage fluid protein. LPS exposure in addition to allergen challenge in the co-administration protocol further increased airway oedema. Therefore, our results indicate that both the timing and dose of LPS exposure are critical for exacerbating airway oedema. This effect may be mediated by increases in LPS binding protein in the lavage fluid, which enhances LPS activation at TLR4 (Dubin *et al*., 1996).

Mucus secretion is a hallmark of asthma exacerbations commonly seen in asthmatics that have died from status asthmaticus (Kuyper *et al*., 2003). LPS alone increases mucus production via goblet cell hyperplasia (Toward and Broadley, 2002), although LPS contamination in commercial Ova during sensitization does not contribute to goblet cell hyperplasia (Tsuchiya *et al*., 2012). In the present study, goblet cell staining increased after two doses of LPS regardless of administration protocol and the presence of Ova. This indicates that the number of LPS exposures is the critical factor in determining an increase in goblet cells. Histological assessment of lung pathology revealed that peribronchiolar inflammation increased following Ova challenge but was not increased further by the addition of LPS exposure, confirming previous results (Tsuchiya *et al*., 2012).

### Sensitivity to inhaled FP

Inhaled corticosteroids have wide ranging effects on the inflammatory and functional responses to allergen that contribute to asthma. In this study, FP significantly attenuated the LAR, AHR and Th2 pulmonary inflammation (IL-13, eosinophilia) in a dose-dependent manner. As previously established, FP demonstrated limited effect on the EAR (Palmqvist *et al*., 2005). Furthermore, this study established that the range of FP activity on allergen-induced responses is relatively narrow, demonstrating full activity at 0.5 mg·mL^−1^ but no activity at 0.1 mg·mL^−1^. The effective doses of FP were then used to determine corticosteroid sensitivity in the LPS/Ova combination models.

Asthma exacerbations contribute to corticosteroid insensitivity (Ito *et al*., 2008). The double LPS exposure co-allergen administration model developed in this study demonstrates several exacerbated features including elongated EAR, a prolonged bronchoconstrictor response to histamine and increased inflammation, with a predominance of neutrophils. The sensitivity of this model to inhaled FP was then established. Contrasting with effects of inhaled FP on allergen alone, the highest doses of FP did not reduce LAR, AHR and cellular inflammation when LPS was added to allergen challenge. Thus, LPS exposure decreases the sensitivity of allergen-induced responses to inhaled FP. In contrast, Ova and LPS combined responses have been reported as partially sensitive to systemically administered dexamethasone (Komlósi *et al*., 2006). Whether this is due to differences in the routes of administration between studies requires investigation.

The functional and inflammatory responses to LPS alone seem to be intrinsically less sensitive to inhaled FP, remaining unchanged in the current study. This has also been observed in several other studies using single and multiple doses of inhaled corticosteroids (Trapp *et al*., 1998; Michel *et al*., 2000). However, the FP insensitivity with allergen and LPS is not simply due to any intrinsic inhaled corticosteroid insensitivity of LPS-induced inflammation as features of allergen-induced inflammation such as eosinophils were also rendered insensitive.

Neutrophils are observed in increased numbers in corticosteroid-insensitive asthmatics (McKinley *et al*., 2008). The neutrophilic nature of the inflammatory response in these people may contribute to corticosteroid insensitivity as neutrophils are less sensitive to corticosteroids then other inflammatory cell types (Strickland *et al*., 2001; Uddin *et al*., 2010). Moreover, they release a range of granule products and inflammatory mediators that increase the oxidative and nitroxic burden on the airways (Ito *et al*., 2008). This can up-regulate glucocorticoid receptor-β, decrease histone deacetylase-2 activity and increase PI3K/Akt pathway activation, all of which can reduce corticosteroid sensitivity (Strickland *et al*., 2001; Ito *et al*., 2008; Mercado *et al*., 2011). Neutrophilia in steroid-insensitive mice is associated with high levels of IL-8 and IL-17 (McKinley *et al*., 2008), which are likely to be important in sustaining the raised neutrophilia observed with allergen and LPS co-administration. Indeed, IL-8 levels were raised significantly above naïve levels by Ova and LPS co-administration.

Corticosteroid insensitivity in asthma lies on a spectrum, with complete resistance rare (Szefler *et al*., 2002). The current study suggests that the insensitivity in the allergen/LPS model may be due to a rightwards shift in the dose–response curve to FP rather than complete resistance. At the highest dose of FP, a non-significant reduction in the LAR was observed. There is also evidence that certain populations of cells such as lymphocytes (Yang *et al*., 2009) may remain steroid sensitive, and here cell-released mediators such as IL-13 were still reduced by FP. The present study revealed non-significant reductions in lymphocyte numbers with FP, suggesting that differentiation of lymphocyte types may reveal steroid-sensitive populations. Finally, FP continued to reduce levels of total protein in lavage fluid with the allergen and LPS combination model, as with allergen alone. However, this effect could be mediated by non-genomic pathways. Corticosteroids decrease uptake of noradrenaline at sympathetic synapses, potentiating noradrenaline's action at α_1_-adrenoceptors, promoting vasoconstriction and thereby decreasing fluid extravasation (Wanner *et al*., 2004). However, as FP was unable to reduce lavage fluid protein induced by LPS alone in the present and previous studies (O'Leary *et al*., 1996), the mechanism behind this effect may be more complicated. Although this model exhibited insensitivity to inhaled FP, we have shown that there is normal sensitivity to systemically administered dexamethasone (Lowe *et al*., 2013).

## Conclusions

This study demonstrated that LPS had dichotomous effects on allergen-induced airway function and inflammation determined by the timing and number of LPS exposures. The ability of LPS to exacerbate airway inflammation, worsen airway function and decrease corticosteroid sensitivity has important implications. The widespread presence of LPS in the environment probably makes it a significant contributor in the development of asthma exacerbations and corticosteroid insensitivity in humans. An increased understanding of how LPS contributes to these processes could lead to novel treatments in an area of unmet clinical need.

## Abbreviations

AHR: airway hyper-responsiveness
EAR: early asthmatic response
FP: fluticasone propionate
LAR: late asthmatic response
Ova: ovalbumin
sG_aw_: specific airway conductance
